# Nomograms for Predicting Specific Distant Metastatic Sites and Overall Survival of Breast Invasive Ductal Carcinoma Patients After Surgery: A Large Population-Based Study

**DOI:** 10.3389/fsurg.2022.779220

**Published:** 2022-03-24

**Authors:** Yuqian Feng, Yiting Zhang, Yuying Xiang, Kaibo Guo, Huimin Jin, Shanming Ruan, Zhuoya Guan

**Affiliations:** ^1^The First Clinical College of Zhejiang Chinese Medical University, Hangzhou, China; ^2^Department of Medical Oncology, The Second Affiliated Hospital of Zhejiang Chinese Medical University, Hangzhou, China; ^3^Department of Medical Oncology, The First Affiliated Hospital of Zhejiang Chinese Medical University, Hangzhou, China; ^4^Department of Mammary Gland, Medical Community of Taizhou Luqiao Traditional Chinese Medicine Hospital, Taizhou, China

**Keywords:** invasive ductal carcinoma, distant metastatic sites, overall survival, nomogram, SEER

## Abstract

**Background:**

Breast cancer (BC) has become the most common malignancy worldwide, accounting for 11.7% of newly diagnosed cancer cases last year. Invasive ductal carcinoma (IDC) is the most common pathological type of BC. However, there were few studies to predict distant metastatic sites and overall survival (OS) of IDC patients.

**Methods:**

Post-operative IDC patients from 2010 to 2016 in the Surveillance, Epidemiology, and End Results (SEER) database were reviewed. Nomograms were developed to predict the specific distant metastatic sites and OS of IDC patients. The performance of nomograms was evaluated with the calibration curves, area under the curve (AUC), and decision curve analysis (DCA). Kaplan-Meier analysis and log-rank tests were used to estimate the survival times of IDC patients with distant metastases.

**Results:**

A total of 171,967 post-operative IDC patients were enrolled in our study. Univariate and multivariate analyses were used to establish the nomograms of significant variables. The AUC of the nomograms for the prediction of liver, lung, bone, and brain metastases was 0.903, 0.877, 0.863, and 0.811, respectively. In addition, the AUC of the nomogram for the prediction of 1-, 3-, and 5-year OS was 0.809, 0.813, 0.787, respectively. Calibration curves and DCA showed good consistency and clinical benefits, respectively.

**Conclusions:**

We constructed new predictive models for liver, lung, brain, bone metastases and 1-, 3-, and 5-year OS in IDC patients. These can help clinicians to individualize the treatment of IDC patients, so that patients can get the more appropriate treatment options.

## Introduction

Breast cancer (BC) is one of the common malignancies, and its incidence is increasing at a rate of 0.5% per year (1). According to the latest global cancer statistics, BC has surpassed lung cancer to become the most commonly diagnosed cancer, with an estimated 2.3 million new cases in 2020 (2). Although the incidence of BC has increased year by year, its death rate has declined, owe to the improvement of diagnostic techniques and the standardization of treatment. Since 1989 to 2018, the death rate in women has dropped by 41% (3). However, for patients with advanced or metastatic BC at the time of diagnosis, the 5-year survival rate was only 23% (4). The management of metastatic BC remains a major challenge.

The TNM staging system is an internationally recognized tool for evaluating tumor prognosis. However, it involves only three prognostic factors, so its accuracy remains to be determined. Similarly, it does not assess the risk of distant metastases from malignancies. Invasive ductal carcinoma (IDC) is the most common pathological type of BC. Therefore, it is very necessary to develop new forecasting tools for it. Huang et al. (5) previously constructed a predictive model for IDC. However, their model only involved bone metastases and did not discuss its differences from the current standard TNM staging system. In addition, we found few other predictive models for IDC prognosis and metastasis.

In this study, nomograms for predicting liver, lung, bone, and brain metastases as well as prognostic nomogram for IDC were constructed and compared with the TNM staging system. An accurate predictive model is needed in order for clinicians to make more accurate judgments and provide personalized treatment strategies for patients.

## Methods

### Patients

**Figure 1:**
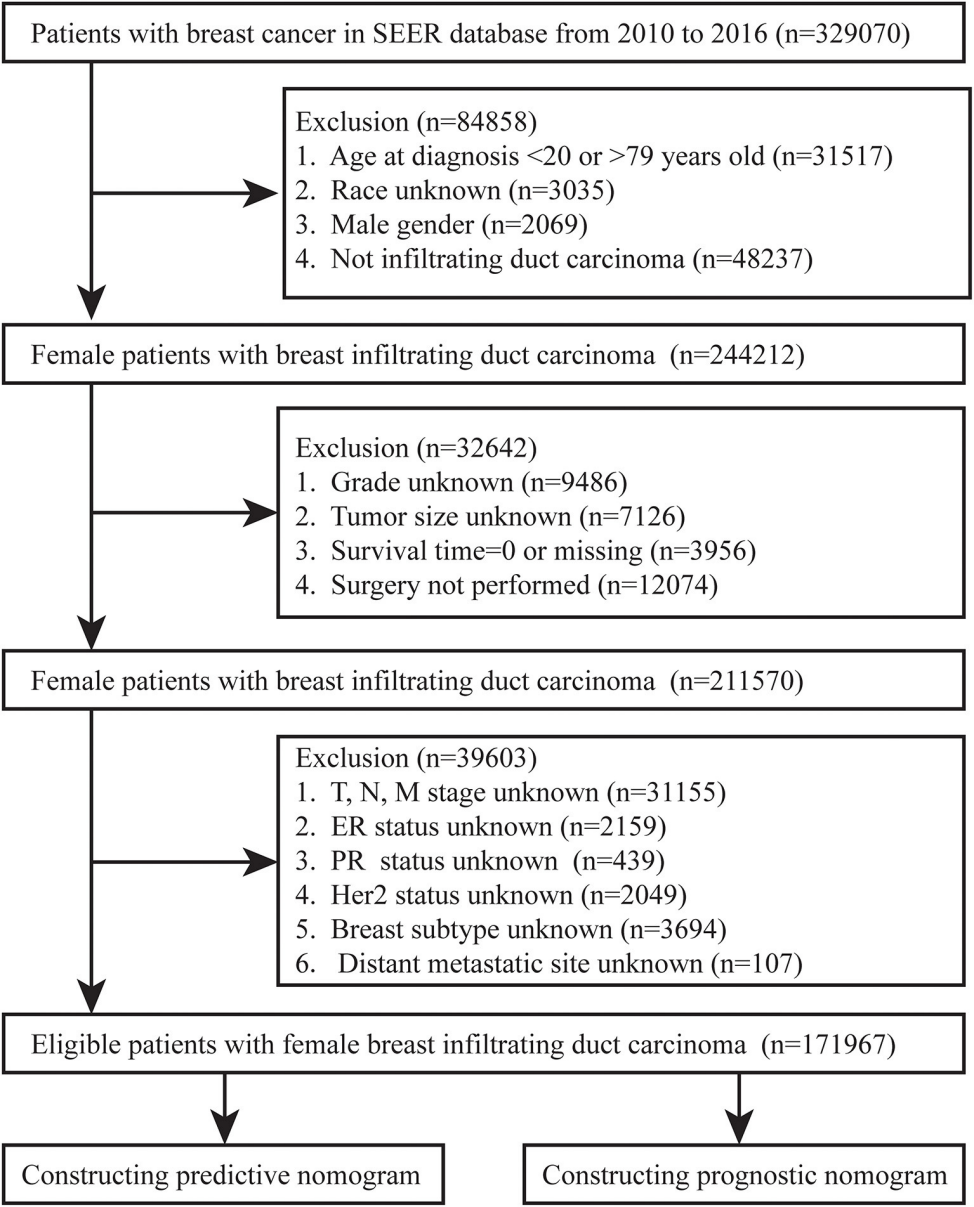
Flowchart of inclusion and exclusion of patients with invasive ductal carcinoma.

We collected BC patients diagnosed between 2010 and 2016 from the Surveillance, Epidemiology, and End Results (SEER) database. The flow chart of inclusion and exclusion of patients was shown in Figure 1. We included female patients with IDC after surgery and excluded patients with unknown variables, such as race, grade, tumor size, TNM stage, and BC subtype. In addition, patients with unknown distant metastatic sites, survival status and time for outcome indicators were excluded. Finally, a total of 17,976 female IDC patients were enrolled in our study.

### Data Collection

Patient demographic variables, including age at diagnosis, marital status, and race, were extracted from the database. Tumor factors, including tumor site, tumor size, grade, T stage, N stage, molecular type and metastatic sites (bone, brain, liver, and lung). Treatment modalities, such as radiotherapy and chemotherapy, as well as survival status and survival time were obtained from the database.

### Development of Prediction and Prognostic Nomograms

All statistical analyses in our research were performed in R software (version 4.1.0). Univariate and multivariate logistic and COX regressions identified independent predictive and prognostic factors for specific distant metastatic site and OS. Odds ratio (OR) and hazard ratio (HR) were used to measure the impact of each independent predictive and prognostic factor on specific distant metastatic sites and OS, respectively. Variables with a *P*-value <0.05 in the multivariate analysis were considered statistically significant. These variables were identified as risk factors and the nomograms were plotted.

### Verification of the Nomograms

The differentiation and calibration abilities of the nomograms were evaluated by receiver operating characteristic (ROC) curves and calibration curves. The area under the ROC curve is represented by the value of the area under curve (AUC), and the closer the AUC value is to 1, the higher the differentiation degree of the model is. The closer the calibration curve is to the reference line in the middle, it represents a higher degree of consistency between the probability of the outcome predicted by the model and the actual observation probability. The clinical benefit of the nomograms was evaluated using decision curve analysis (DCA). The prediction error curve of the model was used to compare the TNM staging system error rate with that of the prognostic nomogram over time. In addition, Kaplan–Meier analysis and log-rank tests were used to estimate the survival times of IDC patients with distant metastases.

## Results

### Patient Characteristics

According to our criteria, a total of 171,967 IDCs were included. Among this group, we found that 93.6% were over 40 years old. Caucasians accounted for 78.5%, which should be related to the selection of patients from the US database. One-third of IDCs occurred in the Upper outer, followed by the Upper inner. More than half of IDCs' tumor size were 1–3 cm, and only 5.9% were larger than 5 cm. Luminal A was the molecular type of more than 70% of the patients, and 12.1% of the patients were triple-negative BC. About half of the patients received chemotherapy or radiation therapy. More than 60% of IDC patients were found to be grade I/ II, T1 or N0. Moreover, most patients do not have lung, liver, brain, or bone metastases at the time of diagnosis. The basic information of the included patients was shown in Table 1.

**Table 1:** Demographics and clinical characteristics of female patients with breast cancer.

**Characteristics**	**Level**	**Number (%)**
Age at diagnosis	20–39	11,042 (6.4)
	40–59	82,147 (47.8)
	60–79	78,778(45.8)
Race	White	134,968 (78.5)
	Black	19,304 (11.2)
	Asian or Pacific Islander	16,549 (9.6)
	American Indian/Alaska Native	1,146 (0.7)
Marital status	Married	101,935 (59.3)
	Other	70,032(40.7)
Site	Lower-inner	9,952 (5.8)
	Lower-outer	13,306 (7.7)
	Upper-inner	22,546 (13.1)
	Upper-outer	61,122 (35.5)
	Nipple-central	7,676 (4.5)
	Other	57,365 (33.4)
Size	≤ 1 cm	44,586 (25.9)
	1–3 cm	96,921(56.4)
	3–5 cm	20,258 (11.8)
	≥5 cm	10,202 (5.9)
Grade	I/II	110,116 (64.0)
	III/IV	61,851 (36.0)
T stage	T1	107,002 (62.2)
	T2	52,779 (30.7)
	T3	8,534 (5.0)
	T4	3,652 (2.1)
N stage	N0	116,322 (67.6)
	N1	41,384 (24.1)
	N2	9,334 (5.4)
	N3	4,927 (2.9)
Liver metastasis	No	171,395 (99.7)
	Yes	572 (0.3)
Lung metastasis	No	171,318 (99.6)
	Yes	649 (0.4)
Bone metastasis	No	170,484 (99.1)
	Yes	1,483 (0.9)
Brain metastasis	No	171,871 (99.9)
	Yes	96 (0.1)
Molecular type	Luminal A	122,758 (71.4)
	Luminal B	20,120 (11.7)
	HER2 enriched	8,320 (4.8)
	Triple negative	20,769 (12.1)
Radiation	No/Unknown	71,527 (41.6)
	Yes	100,440 (58.4)
Chemotherapy	No/Unknown	90,569 (52.7)
	Yes	81,398 (47.3)

### Risk Factors for IDC Patients

Univariate logistic regression indicated that the 11 variables included were all associated with lung, liver, and bone metastases, including age at diagnosis, race, marital status, tumor site, size, grade, T stage, N stage, molecular type, chemotherapy, and radiation. However, marital status did not play a role in the risk factors for brain metastases (Table 2).

**Table 2:** Univariate logistic regression for the presence of different metastatic sites at diagnosis of breast cancer.

	**Liver metastasis**		**Lung metastasis**		**Bone metastasis**		**Brain metastasis**	
**Characteristics**	**OR (95% CI)**	***P*** **-value**	**OR (95% CI)**	***P*** **-value**	**OR (95% CI)**	***P*** **-value**	**OR (95% CI)**	***P*** **-value**
**Age at diagnosis**								
20–39	1		1		1		1	
40–59	0.486 (0.383–0.623)	<0.001	0.593 (0.455–0.785)	<0.001	0.527 (0.449–0.622)	<0.001	0.685 (0.364–1.433)	0.275
60–79	0.316 (0.245–0.411)	<0.001	0.660 (0.507–0.872)	<0.01	0.425 (0.361–0.504)	<0.001	0.490 (0.252–1.046)	<0.05
**Race**								
White	1		1		1		1	
Black	1.786 (1.435–2.202)	<0.001	2.040 (1.674–2.470)	<0.001	1.513 (1.313–1.737)	<0.001	1.723 (0.981–2.858)	<0.05
Asian or Pacific Islander	0.921 (0.672–1.232)	0.594	1.006 (0.754–1.316)	0.966	0.776 (0.632–0.941)	<0.05	1.064 (0.495–2.019)	0.862
American Indian/Alaska Native	0.849 (0.210–2.218)	0.778	1.821 (0.778–3.557)	0.117	1.155 (0.597–1.992)	0.636	1.707 (0.967–7.712)	0.596
**Marital status**								
Married	1		1		1		1	
Other	1.312 (1.112–1.546)	<0.01	1.676 (1.436–1.957)	<0.001	1.423 (1.285–1.576)	<0.001	1.181 (0.786–1.764)	0.418
**Site**								
Lower-inner	1		1		1		1	
Lower-outer	1.385 (0.812–2.431)	0.241	1.052 (0.671–1.669)	0.827	1.027 (0.750–1.413)	0.868	0.374 (0.100–1.187)	0.108
Upper-inner	0.794 (0.465–1.397)	0.409	0.648 (0.415–1.024)	0.058	0.803 (0.597–1.088)	0.15	0.221 (0.059–0.700)	<0.05
Upper-outer	1.352 (0.872–2.219)	0.202	0.967 (0.675–1.432)	0.859	0.994 (0.773–1.299)	0.963	0.671 (0.326–1.563)	0.312
Nipple-central	2.471 (1.454–4.329)	<0.01	1.420 (0,878–2.305)	0.152	1.967 (1.446–2.693)	<0.001	0.324 (0.049–1.293)	0.154
Other	2.392 (1.561–3.892)	<0.001	1.630 (1.150–2.393)	<0.01	1.801 (1.412–2.338)	<0.001	0.976 (0.487–2.237)	0.949
**Size**								
≤ 1 cm	1		1		1		1	
1–3 cm	4.351 (2.755–7.324)	<0.001	3.158 (2.046–5.136)	<0.001	5.177 (3.838–7.176)	<0.001	5.675 (2.053–23.514)	<0.01
3–5 cm	20.208 (12.782–34.049)	<0.001	18.279 (11.906–29.613)	<0.001	23.426 (17.343–32.507)	<0.001	17.627 (6.161–74.182)	<0.001
≥5 cm	54.570 (34.759–91.483)	<0.001	66.726 (44.010–107.106)	<0.001	54.509 (40.414–75.547)	<0.001	46.760 (16.745–194.636)	<0.001
**Grade**								
I/II	1		1		1		1	
III/IV	5.082 (4.227–6.145)	<0.001	4.458 (3.766–5.297)	<0.001	2.261 (2.040–2.507)	<0.001	5.061 (3.255–8.136)	<0.001
**T stage**								
T1	1		1		1		1	
T2	6.186 (4.790–8.081)	<0.001	8.100 (6.043–1.105)	<0.001	7.391 (6.288–8.733)	<0.001	6.242 (3.433–12.137)	<0.001
T3	19.474 (14.582–26.163)	<0.001	30.240 (22.060–42.036)	<0.001	17.833 (14.737–21.621)	<0.001	10.622 (4.663–23.758)	<0.001
T4	62.766 (47.717–83.282)	<0.001	154.034 (115.425–209.507)	<0.001	69.187 (57.930–82.927)	<0.001	72.751 (39.061–143.726)	<0.001
**N stage**								
N0	1		1		1		1	
N1	7.467 (5.858–9.605)	<0.001	6.728 (5.416–8.414)	<0.001	7.181 (6.169–8.387)	<0.001	5.329 (3.095–9.477)	<0.001
N2	19.165 (14.639–25.216)	<0.001	15.887 (12.408–20.388)	<0.001	18.580 (15.688–22.042)	<0.001	9.853 (4.928–19.346)	<0.001
N3	34.207 (25.999–45.200)	<0.001	26.770 (20.727–34.612)	<0.001	38.290 (32.345–45.411)	<0.001	32.473 (18.047–59.511)	<0.001
**Molecular type**								
Luminal A	1		1		1		1	
Luminal B	4.021 (3.239–4.976)	<0.001	2.618 (2.096–3.248)	<0.001	1.792 (1.562–2.049)	<0.001	2.348 (1.254–4.167)	<0.01
HER2 enriched	7.388 (5.822–9.319)	<0.001	5.259 (4.139–6.626)	<0.001	1.697 (1.380–2.065)	<0.001	4.924 (2.528–8.978)	<0.001
Triple negative	3.246 (2.581–4.064)	<0.001	3.758 (3.095–4.550)	<0.001	1.063 (0.898–1.249)	0.471	4.400 (2.699–7.092)	<0.001
**Radiation**								
Yes	1		1		1		1	
No/Unknown	2.541 (2.144–3.021)	<0.001	2.401 (2.049–2.820)	<0.001	1.293 (1.167–1.432)	<0.001	0.260 (0.144–0.437)	<0.001
**Chemotherapy**								
Yes	1		1		1		1	
No/Unknown	0.176 (0.140–0.218)	<0.001	0.293 (0.244–0.349)	<0.001	0.357 (0.318–0.399)	<0.001	0.207 (0.120–0.338)	<0.001

*OR, odds ratio; CI, confidence interval*.

The statistically significant variables of univariate logistic regression were included in multivariate logistic regression for further analysis. We found that age affected liver, lung, and bone metastasis in IDC patients, but had no effect on brain metastasis. Ethnic differences had little effect on distant migration. Marital status was not a risk factor for distant metastasis. The site of tumor was not associated with liver metastasis, but IDC in nipple-central was less prone to lung metastasis, in upper-outer was less prone to bone metastasis, and IDC patients who developed in the nipple-central and upper-outer region were less likely to develop brain metastasis. The size of the tumor was one of the risk factors for distant metastasis. The larger the tumor, the higher the risk of distant metastasis. Similarly, grade III /IV, site of lymph node metastasis were risk factors for distant metastasis. Luminal B, HER2 Enriched, and Triple Negative were more prone to distant metastasis than Luminal A (Table 3).

**Table 3:** Multivariate logistic regression for the presence of different metastatic sites at diagnosis of breast cancer.

	**Liver metastasis**		**Lung metastasis**		**Bone metastasis**		**Brain metastasis**	
**Characteristics**	**OR (95% CI)**	***P*** **-value**	**OR (95% CI)**	***P*** **-value**	**OR (95% CI)**	***P*** **-value**	**OR (95% CI)**	***P*** **-value**
**Age at diagnosis**								
20–39	1		1		1		1	
40–59	0.776 (0.608–1.002)	<0.05	0.944 (0.719–1.257)	0.684	0.766 (0.649–0.909)	<0.01	1.090 (0.575–2.289)	0.806
60–79	0.710 (0.544–0.935)	<0.05	1.378 (1.044–1.844)	<0.05	0.741 (0.621–0.887)	<0.01	1.041 (0.523–2.259)	0.913
**Race**								
White	1		1		1		1	
Black	1.119 (0.890–1.395)	0.327	1.187 (0.961–1.457)	0.107	1.054 (0.906–1.220)	0.491	0.980 (0.553–1.645)	0.941
Asian or Pacific Islander	0.764 (0.554–1.027)	0.085	0.925 (0.688–1.220)	0.591	0.688 (0.558–0.839)	<0.001	1.029 (0.477–1.965)	0.935
American Indian/Alaska Native	0.800 (0.197–2.111)	0.702	1.823 (0.767–3.642)	0.125	1.0372 (0.528–1.823)	0.907	1.400 (0.079–6.484)	0.741
**Marital status**								
Married	1		1		1		–	
Other	1.005 (0.845–1.195)	0.952	1.111 (0.942–1.310)	0.210	1.105 (0.991–1.231)	0.072	–	–
**Site**								
Lower-inner	1		1		1		1	
Lower-outer	1.128 (0.657–1.993)	0.669	0.897 (0.565–1.441)	0.648	0.815 (0.591–1.130)	0.216	0.328 (0.0872–1.047)	0.070
Upper-inner	0.879 (0.511–1.555)	0.646	0.723 (0.459–1.155)	0.168	0.867 (0.641–1.183)	0.361	0.242 (0.064–0.770)	<0.05
Upper-outer	1.032 (0.660–1.703)	0.897	0.757 (0.522–1.133)	0.157	0.752 (0.581–0.989)	<0.05	0.533 (0.257–1.249)	0.114
Nipple-central	1.264 (0.735–2.236)	0.407	0.591 (0.360–0.975)	<0.05	0.885 (0.644–1.224)	0.456	0.187 (0.028–0.756)	<0.05
Other	1.379 (0.892–2.259)	0.173	0.852 (0.593–1.265)	0.405	1.033 (0.803–1.350)	0.808	0.652 (0.321–1.509)	0.273
**Size**								
≤ 1 cm	1		1		1		1	
1–3 cm	1.447 (0.875–2.521)	0.168	0.992 (0.598–1.703)	0.974	1.866 (1.341–2.653)	<0.001	2.050 (0.645–9.085)	0.271
3–5 cm	2.462 (1.413–4.486)	<0.01	1.897 (1.095–3.397)	<0.05	3.162 (2.205–4.619)	<0.001	2.078 (0.577–10.028)	0.302
≥5 cm	3.107 (1.675–6.023)	<0.001	2.942 (1.650–5.437)	<0.001	4.301 (2.867–6.565)	<0.001	2.929 (0.738–15.422)	0.157
**Grade**								
I/II	1		1		1		1	
III/IV	1.786 (1.457–2.199)	<0.001	1.536 (1.269–1.866)	<0.001	1.132 (1.007–1.272)	<0.05	1.986 (1.198–3.387)	<0.01
**T stage**								
T1	1		1		1		1	
T2	1.779 (1.296–2.465)	<0.001	3.360 (2.320–4.961)	<0.001	2.910 (2.386–3.565)	<0.001	2.948 (1.438–6.455)	<0.01
T3	2.172 (1.321–3.548)	<0.05	4.642 (2.848–7.601)	<0.001	2.646 (1.918–3.643)	<0.001	2.249 (0.677–7.253)	0.178
T4	6.490 (4.215–9.921)	<0.001	23.886 (15.422–37.261)	<0.001	10.674 (8.131–13.987)	<0.001	13.150 (5.007–34.296)	<0.001
**N stage**								
N0	1		1		1		1	
N1	3.591 (2.774–4.689)	<0.001	2.859 (2.257–3.643)	<0.001	3.866 (3.277–4.574)	<0.001	2.586 (1.424–4.846)	<0.01
N2	6.513 (4.845–8.794)	<0.001	4.058 (3.074–5.368)	<0.001	6.985 (5.773–8.467)	<0.001	3.137 (1.468–6.643)	<0.01
N3	7.979 (5.864–10.898)	<0.001	4.274 (3.192–5.730)	<0.001	11.366 (9.353–13.836)	<0.001	7.081 (3.552–14.390)	<0.001
**Molecular type**								
Luminal A	1		1		1		1	
Luminal B	2.091 (1.669–2.612)	<0.001	1.469 (1.163–1.846)	<0.01	1.103 (0.953–1.274)	0.183	1.354 (0.707–2.474)	0.339
HER2 enriched	2.741 (2.124–3.521)	<0.001	1.996 (1.539–2.571)	<0.001	0.775 (0.622–0.956)	<0.05	2.060 (1.021–3.937)	<0.05
Triple negative	1.429 (1.114–1.826)	<0.01	1.704 (1.369–2.116)	<0.001	0.590 (0.492–0.705)	<0.001	2.161 (1.257–3.708)	<0.01
**Radiation**								
Yes	1		1		1		1	
No/Unknown	2.897 (2.428–3.465)	<0.001	2.563 (2.167–3.037)	<0.001	1.342 (1.204–1.497)	<0.001	0.247 (0.135–0.422)	<0.001
**Chemotherapy**								
Yes	1		1		1		1	
No/Unknown	0.748 (0.583–0.952)	<0.05	1.181 (0.958–1.451)	0.115	1.479 (1.291–1.693)	<0.001	1.446 (0.762–2.628)	0.241

*OR, odds ratio; CI, confidence interval*.

In Table 4, we found that IDC patients aged 40–59 years had a lower risk of death (HR: 0.927, 95%CI: 0.862–0.997). The risk of death for blacks was 1.281 times that for whites. Other patients (including divorced, widowed, single, etc.) had a higher risk of death than married patients (HR: 1.358, 95%CI: 1.307–1.411). IDC in the Upper-inner and Lower-outer regions had a relatively low risk of death. Triple negative IDC patients had the highest risk of death among all molecular subtypes (HR: 2.392, 95%CI: 2.276–2.515). Of course, patients with distant metastases also had a higher risk of death than those without metastases.

**Table 4:** Univariate and multivariate Cox regression for overall survival based on different metastatic sites of breast cancer.

	**Univariate analyses**		**Multivariate analyses**	
**Characteristics**	**HR (95% CI)**	***P*** **-value**	**HR (95% CI)**	***P*** **-value**
**Age at diagnosis**				
20–39	1		1	
40–59	0.642 (0.597–0.690)	<0.001	0.927 (0.862–0.997)	<0.05
60–79	0.922 (0.859–0.989)	<0.05	1.568 (1.458–1.686)	<0.001
**Race**				
White	1		1	
Black	1.896 (1.808–1.988)	<0.001	1.281 (1.219–1.346)	<0.001
Asian or Pacific Islander	0.658 (0.607–0.714)	<0.001	0.681 (0.628–0.739)	<0.001
American Indian/Alaska Native	1.459 (1.199–1.776)	<0.001	1.236 (1.016–1.504)	<0.05
**Marital status**				
Married	1		1	
Other	1.693 (1.631–1.757)	<0.001	1.358 (1.307–1.411)	<0.001
**Site**				
Lower-inner	1		1	
Lower-outer	0.989 (0.890–1.099)	0.834	0.897 (0.807–0.997)	<0.05
Upper-inner	0.899 (0.816–0.991)	<0.05	0.933 (0.847–1.029)	0.163
Upper-outer	0.994 (0.912–1.083)	0.890	0.866 (0.795–0.944)	<0.01
Nipple-central	1.407 (1.261–1.572)	<0.001	0.991 (0.887–1.107)	0.868
Other	1.186 (1.090–1.291)	<0.001	0.928 (0.852–1.011)	0.087
**Size**				
≤ 1 cm	1		1	
1–3 cm	2.215 (2.074–2.364)	<0.001	1.439 (1.339–1.547)	<0.001
3–5 cm	5.804 (5.409–6.229)	<0.001	2.075 (1.895–2.273)	<0.001
≥5 cm	10.386 (9.658–11.169)	<0.001	2.060 (1.795–2.363)	<0.001
**Grade**				
I/II	1		1	
III/IV	3.006 (2.893–3.124)	<0.001	1.753 (1.675–1.835)	<0.001
**T stage**				
T1	1		1	
T2	2.821 (2.702–2.946)	<0.001	1.435 (1.356–1.519)	<0.001
T3	5.922 (5.576–6.289)	<0.001	1.977 (1.732–2.256)	<0.001
T4	12.063 (11.294–12.883)	<0.001	2.790 (2.505–3.107)	<0.001
**N stage**				
N0	1		1	
N1	2.227 (2.131–2.328)	<0.001	1.785 (1.700–1.873)	<0.001
N2	4.722 (4.462–4.997)	<0.001	3.013 (2.827–3.212)	<0.001
N3	8.644 (8.147–9.172)	<0.001	4.346 (4.059–4.652)	<0.001
**Molecular type**				
Luminal A	1		1	
Luminal B	1.141 (1.069–1.217)	<0.001	0.821 (0.768–0.878)	<0.001
HER2 enriched	1.895 (1.755–2.046)	<0.001	1.058 (0.976–1.147)	0.169
Triple negative	3.416 (3.273–3.565)	<0.001	2.392 (2.276–2.515)	<0.001
**Radiation**				
Yes	1		1	
No/Unknown	1.553 (1.496–1.612)	<0.001	1.497 (1.441–1.555)	<0.001
**Chemotherapy**				
Yes	1		1	
No/Unknown	0.557 (0.536–0.579)	<0.001	1.483 (1.415–1.555)	<0.001
**Liver metastasis**				
No	1		1	
Yes	13.780 (12.300–15.430)	<0.001	2.356 (2.070–2.680)	<0.001
**Lung metastasis**				
No	1		1	
Yes	14.01 (12.630–15.540)	<0.001	1.725 (1.532–1.943)	<0.001
**Bone metastasis**				
No	1		1	
Yes	9.484 (8.769–10.260)	<0.001	2.196 (1.996–2.415)	<0.001
**Brain metastasis**				
No	1		1	
Yes	25.880 (20.650–32.440)	<0.001	4.628 (3.652–5.865)	<0.001

*HR, hazard ratio; CI, confidence interval*.

### Nomograms Development and Validation

**Figure 2:**
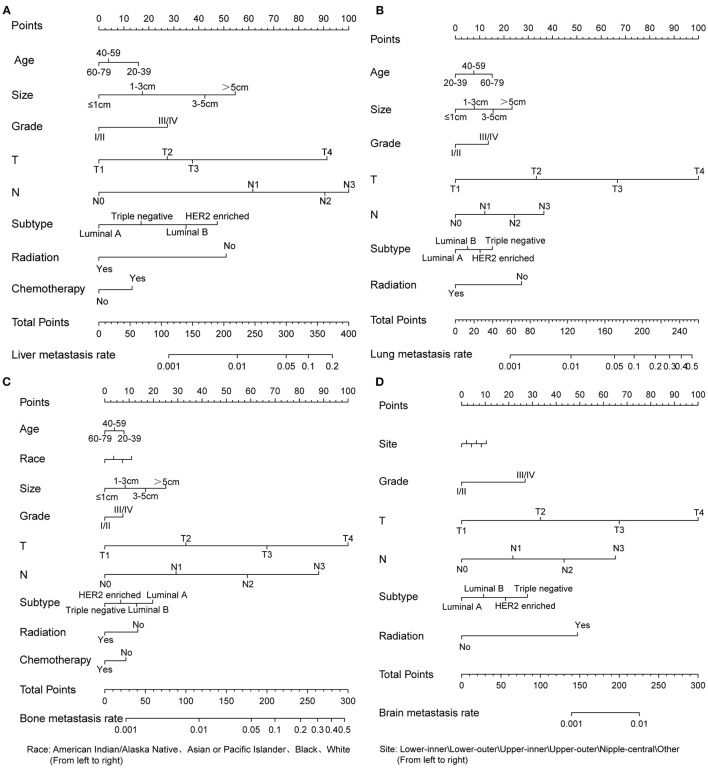
Four nomograms to predict the probability of liver **(A)**, lung **(B)**, bone **(C)**, and brain **(D)** metastases in patients with invasive ductal carcinoma.

**Figure 3:**
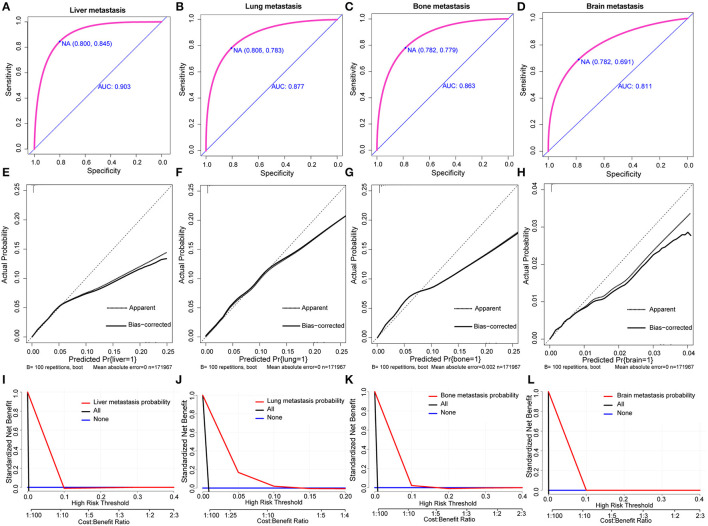
AUC values of ROC predicted liver **(A)**, lung **(B)**, bone **(C)**, and brain **(D)** metastasis rates of Nomogram. The calibration curve of predictive nomograms for predicting invasive ductal carcinoma patients' liver **(E)**, lung **(F)**, bone **(G)**, and brain **(H)** metastasis rates. Decision curve analysis of the predictive nomogram for predicting liver **(I)**, lung **(J)**, bone **(K)**, and brain **(L)** metastasis.

Based on multivariate logistic regression, we constructed the nomograms for predicting liver, lung, bone, and brain metastasis, respectively, as shown in Figure 2. It could be seen that T and N stage have great influence on distant metastasis in different parts. Different sites of metastasis have their own influencing factors. Tumor size and subtype had a great influence on liver metastasis, and radiotherapy had a certain effect on liver, lung and brain metastasis. The AUC of the nomograms for the prediction of liver, lung, bone, and brain metastases were 0.903, 0.877, 0.863, and 0.811, respectively (Figures 3A–D), exhibiting good discrimination. In addition, the solid lines of the calibration curves approach at a 45°, suggesting accurate prediction by these four models (Figures 3E–H). In addition, the DCA also proved the value of the four models. The net benefit of our predictive models were larger than that in other scenarios (all screening or none-screening) in a wide range of threshold probabilities (Figures 3I–L).

**Figure 4:**
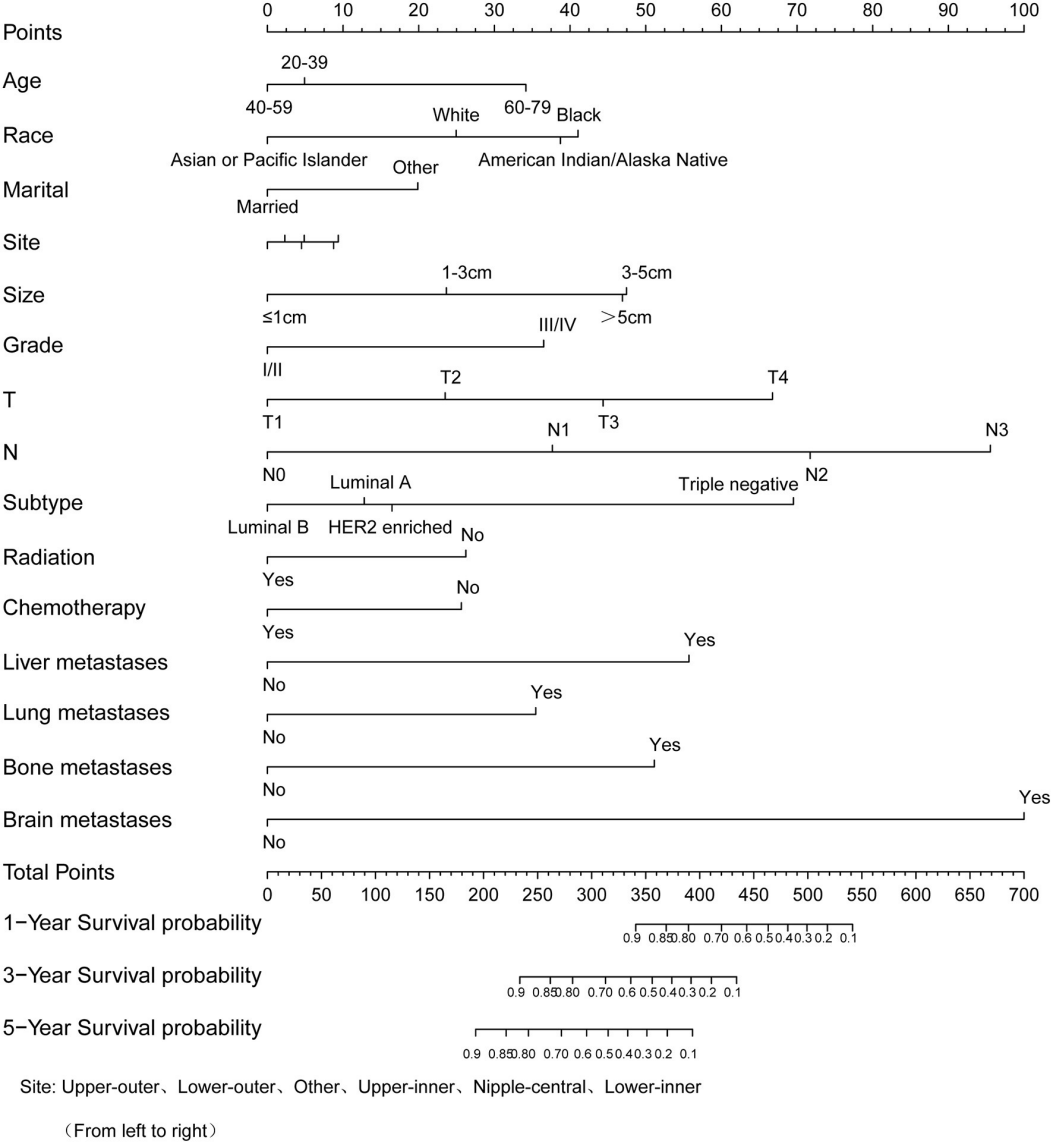
Nomogram to predict the survival probability of 1-, 3-, 5-year in patients with invasive ductal carcinoma.

**Figure 5:**
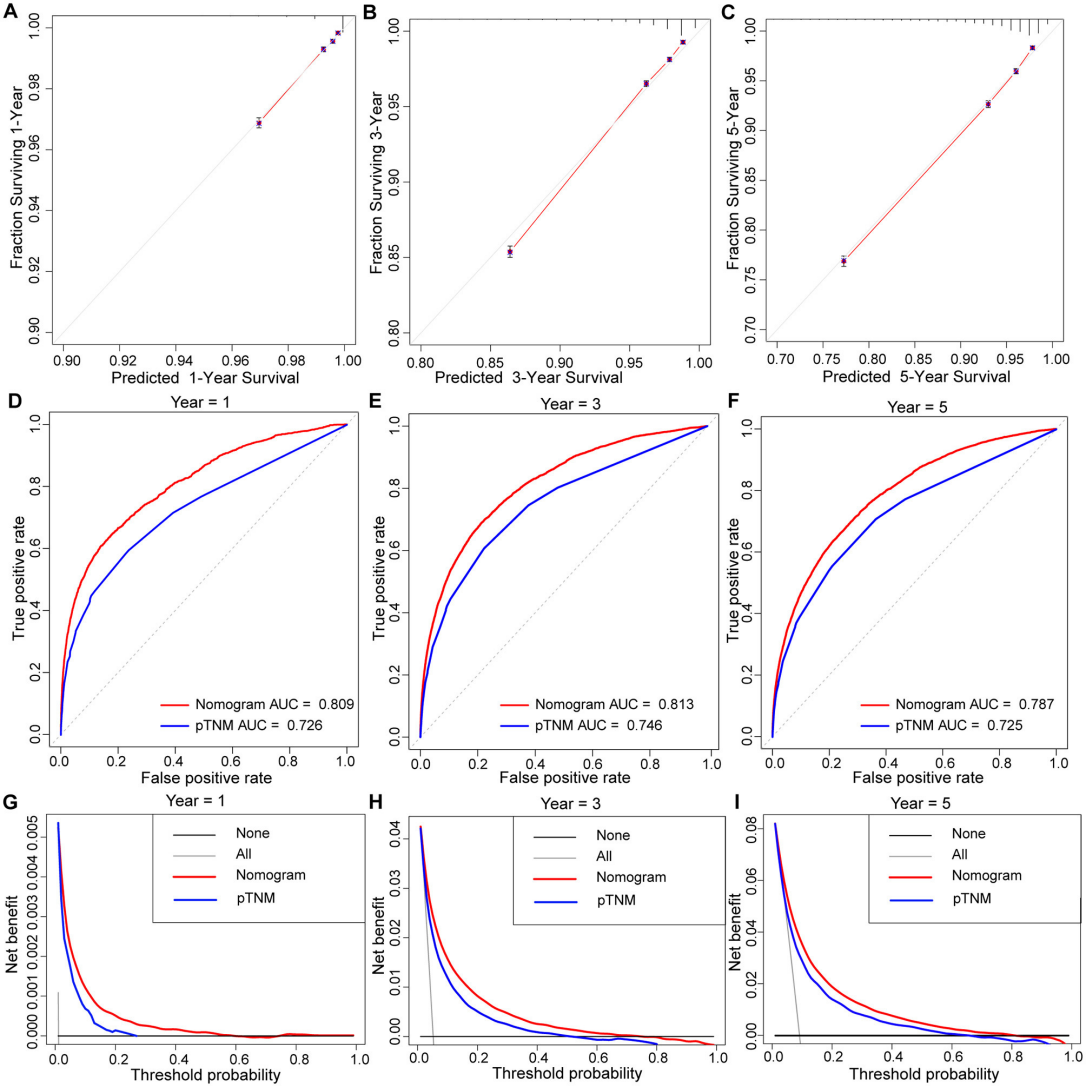
The calibration curve of prognostic nomogram for predicting invasive ductal carcinoma patients' 1-year **(A)**, 3-year **(B)**, 5-year **(C)** survival probability. AUC values of ROC predicted 1-year **(D)**, 3-year **(E)**, 5-year **(F)** survival probability of Nomogram and TNM staging systerm. Decision curve analysis of the prognostic nomogram and TNM staging system for predicting 1-year **(G)**, 3-year **(H)**, 5-year **(I)** survival probability.

**Figure 6:**
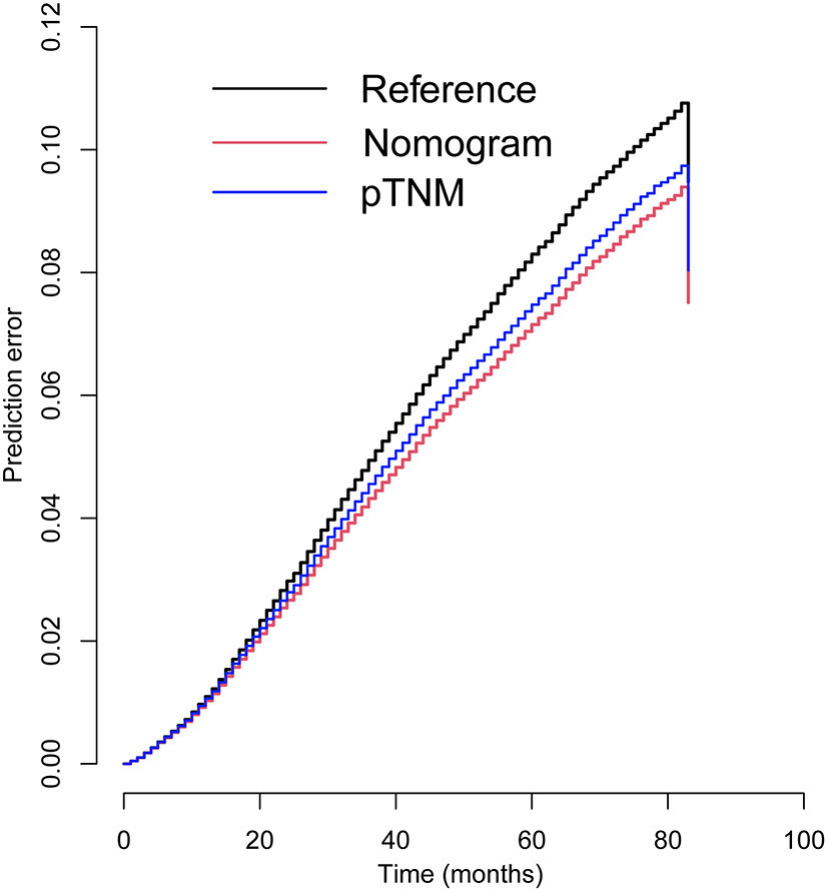
Prediction error curves for prognostic nomogram and TNM stage to predict patients' overall survival.

Similarly, based on multivariate Cox regression, we established 1-, 3-, 5-year prognosis nomograms for IDC patients (Figure 4). It could be seen that race, tumor size, T stage, N stage and subtype had great influence on the survival of BC patients, and N stage was the most obvious one. Among the different distant sites of metastasis, brain metastasis had the greatest impact on the prognosis of patients, followed by liver metastasis and bone metastasis. The calibration curves of 1-, 3-, and 5-year all fit well with the reference line (Figures 5A–C). The AUC of the nomograms for the prediction of 1-, 3-, and 5-year was 0.809, 0.813, 0.787, respectively (Figures 5D–F). DCA also showed the good clinical implementation significance in predictive survival rate (Figures 5G–I). We also compared the nomogram with the recognized TNM staging system, and both the ROC curves and DCA showed that the nomogram was superior to TNM staging system. The *P*-values of ROC curves for 1-, 3-, and 5-year nomogram compared with TNM staging system are calculated and they are all <0.001. For 1-, 3-, and 5-year nomogram, Δnet benefit (ΔNB) of the nomogram vs. the baseline model is 0.000471, 0.00399, and 0.00718, and the test tradeoff is 212.5 (=1/0.00471), 25.1 (=1/0.0399), and 13.9 (=1/0.0718), respectively. If we consider it acceptable to subject 213, 25, or 14 patients to our nomogram to detect one patient's 1-, 3-, or 5-year survival compared with the model with TNM stage, the utility of the extended model is worth the cost of our nomogram. Meanwhile, we also included the prediction error model, and compared the nomogram with the TNM staging system, it was found that the error rate of the nomogram was lower (Figure 6).

### Survival Analysis in IDC Patients With Distant Metastasis

**Figure 7:**
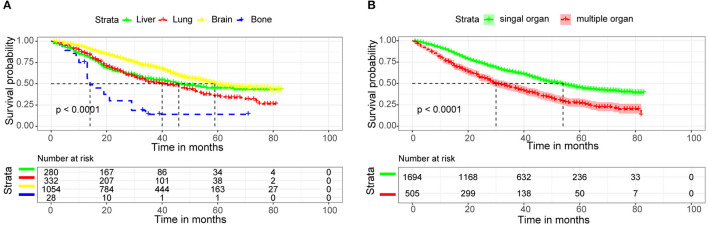
Kaplan-Meier survival curves for distant metastatic site **(A)**, Number of metastatic organs **(B)** in invasive ductal carcinoma patients.

We used Kaplan-Meier analysis and log-rank tests to analyze the survival rates of different metastatic sites and single or multiple organ metastases. In Figure 7A, we found that patients with bone metastases had the lowest survival rate, with the median survival time of <20 months. While patients with brain metastases had the highest survival rate, with the median survival time of nearly 60 months. Median survival time was longer than 50 months for patients with single organ metastases and only 30 months for patients with multiple organ metastases (Figure 7B).

## Discussion

The incidence of BC has been increasing year by year. In 2020, BC has become the most common cancer in the world. Currently, the 5-year OS rate for BC is >90%. There was a significant improvement in survival rate compared to earlier times, a trend that can be explained by early diagnosis and more appropriate treatment regimens (6, 7). Even so, the 5-year survival rate for women with advanced or metastatic BC is only 26% (4). IDC is the most common pathological type of BC. Surgical excision is the main method for ocular treatment of IDC. Therefore, it is particularly important to understand the risk factors of IDC metastasis after operation.

The nomogram is established by incorporating a variety of factors that have an impact on the prognosis and survival. These factors can be quantified and the results can be objectively displayed, so as to accurately predict the prognosis and survival of patients. Compared with TNM staging, the nomogram can be more specific and detailed to estimate the individual risk according to the characteristics of patients and diseases. In oncology, the nomogram has potential to impact all aspects of cancer care. Preoperative nomograms can predict patients' positive surgical margins and lymph node metastasis, thus facilitating clinicians to choose a more appropriate surgical approach. Postoperative nomograms can predict postoperative recurrence, adjuvant therapy effect and survival, so that clinicians can choose a more appropriate treatment plan for patients (8).

In this study, we used the nomograms to construct the prediction model of IDC patients' liver, lung, bone and brain metastases and the OS rates of IDC patients at 1-, 3-, and 5-year, respectively. In addition, we have verified the nomograms with calibration curves, ROC curves and DCAs, which have a good ability of discrimination and clinical benefits. At the same time, we compare the prognostic nomograms with the existing TNM staging system to obtain more objective results.

BC mainly occurs in the age range of 50 to 69 years. This may be linked to rising rates of obesity and menopausal hormone use among older women. A previous study found that women who took hormones had a 26% increased risk of BC (9). And women with a higher waist-to-hip ratio had a 68% increased risk of dying from BC (10). Young age is associated with the risk of distant metastasis of BC, but its effect on survival remains controversial. Previous studies have shown a significant reduction in the risk of distant metastases in patients over 40 or 45 years of age (11, 12). The protective effect of old age on tumor progression may be related to the changes of host immune system or tumor microenvironment caused by age (13). However, poor baseline conditions, postoperative complications, or inability to tolerate treatment can all affect OS in older patients.

Overall, 71.4% of IDC cases were HR+/HER2– (luminal A), 12.1% were HR–/HER2– (triple negative), 11.7% were HR+/HER2+ (luminal B), and 4.8% were HR–/HER2+ (HER2-enriched). In our study, the influence of molecular subtypes on metastatic sites did not show a significant pattern, but in terms of prognosis, OS was significantly reduced in triple negative and HER2-enriched BC patients. It has been reported that tumor-infiltrating lymphocytes (TILs) densities in HER2-enriched and triple negative BCs is significantly higher than those in luminal A BCs, which is associated with patient prognosis (14, 15). According to reports, patients with TILs > 10% in early triple negative BC had an increased rate of local recurrence compared to patients with low TILs (16). Molecular subtypes were associated with the distribution of ethnicity, with black women having the lowest rate of luminal A and the highest rate of triple negative (6). A study found that among patients diagnosed with small breast tumors ( ≤ 2.0 cm) between 2004 and 2011, black women were more likely to develop lymph node metastases (24% vs. 18%, respectively) than white women (17). Of course, poverty, low education, and a lack of advanced medical equipment and methods all contribute to the higher mortality rate among black women (18, 19).

A previous study from Germany demonstrated that patients with advanced BC (T1/2N+, T3/4N0, T3/4N+) who underwent R0 resection had ~30% higher rates of local recurrence and distant metastasis, and 23% higher rates in high-grade patients, which were 3–4 times higher than those in early and low-grade BC patients, respectively (20). Patients with BC who were treated do have a better prognosis than those who were not. However, for distant metastases, the advantage of chemotherapy is less than that of radiotherapy, which may be related to the fact that BC patients are more likely to receive endocrine therapy. However, our study did not cover this aspect.

Bone metastasis is the main metastatic site of BC, accounting for 50% of all metastatic sites (21). Most BC patients with bone metastases receive palliative treatment, with a median survival of only 2–3 years (22, 23). Another study has shown that HR+/HER2- BC patients had a higher risk of bone metastasis (24). This is similar to our findings. Similarly, the number of organs transferred has a significant effect on survival time. The patients with higher number of metastatic sites always have worse prognosis. Patients with four sites of metastasis have a 2.2 times greater risk of dying than those with only one site of metastasis (25).

There are some shortcomings we cannot avoid in the present study. First, some key information (such as endocrine therapy, biomarker expression states) were not found in the database, which would affect the accuracy of our study. Second, the small number of patients with brain metastasis may affect the accuracy of prediction model. Third, our study is a retrospective study, using internal verification methods, and has not been verified in real world. Fourth, the data was only collected from the US database, so the nomograms we constructed may not be suitable for global patients.

## Conclusions

In conclusion, we constructed new nomograms to predict metastatic sites and OS in IDC patients. This can be used to help clinicians individualize treatment for IDC patients and select more appropriate treatment options. However, the nomograms still requires a large amount of clinical data to validate.

## Data Availability Statement

Publicly available datasets were analyzed in this study. This data can be found here: https://seer.cancer.gov/.

## Author Contributions

YF conceived and designed the study, performed the study, analyzed the data, prepared figures and/or tables, and authored or reviewed drafts of the paper. YZ, YX, HJ, and KG conceived and designed the study, performed the study, and analyzed the data. SR performed the study and authored or reviewed drafts of the paper. ZG conceived and designed the study, performed the study, authored or reviewed drafts of the paper, and approved the final draft. All authors contributed to the article and approved the submitted version.

## Funding

This work was supported by National Natural Science Foundation of China (81573902), China Postdoctoral Science Foundation (2017M612040, 2018T110610), Ten Thousand Talents Program of Zhejiang Province (SR, no. 2019-97, http://www.zjzzgz.gov.cn/); Research Project of Zhejiang Chinese Medical University (2021 JKZKTS043B).

## Conflict of Interest

The authors declare that the research was conducted in the absence of any commercial or financial relationships that could be construed as a potential conflict of interest.

## Publisher's Note

All claims expressed in this article are solely those of the authors and do not necessarily represent those of their affiliated organizations, or those of the publisher, the editors and the reviewers. Any product that may be evaluated in this article, or claim that may be made by its manufacturer, is not guaranteed or endorsed by the publisher.
